# Revision of the velvet spiders (Araneae, Eresidae) with a new record from South Korea

**DOI:** 10.3897/BDJ.13.e165869

**Published:** 2025-08-25

**Authors:** Sue Yeon Lee, Chang Moon Jang, Jung Sun Yoo, Yeong-Seok Jo, Seung Tae Kim

**Affiliations:** 1 R&D Center, Cellcuratio Co., Ltd., Innobiz park, 1646 Yuseongdae-ro, Yuseong-gu, Daejeon, Republic of Korea R&D Center, Cellcuratio Co., Ltd., Innobiz park, 1646 Yuseongdae-ro, Yuseong-gu Daejeon Republic of Korea; 2 Diversity Conservation Research Department, Nakdonggang National Institute of Biological Resources, Ministry of Environment, Sangju, Republic of Korea Diversity Conservation Research Department, Nakdonggang National Institute of Biological Resources, Ministry of Environment Sangju Republic of Korea; 3 Wildlife Quarantine Center, National Institute of Wildlife Disease Control and Prevention, Ministry of Environment, Incheon, Republic of Korea Wildlife Quarantine Center, National Institute of Wildlife Disease Control and Prevention, Ministry of Environment Incheon Republic of Korea; 4 Department of Biology Education, Daegu University, Gyeongsan, Republic of Korea Department of Biology Education, Daegu University Gyeongsan Republic of Korea; 5 Life and Environment Research Institute, Konkuk University, Seoul, Republic of Korea Life and Environment Research Institute, Konkuk University Seoul Republic of Korea

**Keywords:** Asia, biodiversity, distribution, ladybird spider, morphology, taxonomy

## Abstract

**Background:**

The genus *Eresus* Walckenaer, 1805, commonly known as the ladybird spiders, comprises 30 species primarily distributed in the Palaearctic, Afrotropical and Oriental Regions as well as South America, with only one species, *E. kollari* Rossi, 1846, recorded from South Korea.

**New information:**

New taxonomic and distribution data on the genus *Eresus* Walckenaer, 1805, are provided. A newly-recorded species, *E. granosus* Simon, 1895, is described along with *E. kollari* Rossi, 1846 with a detailed description, illustrations and ecological photographs from South Korea.

## Introduction

The spider family Eresidae C. L. Koch, 1845, commonly known as velvet spiders, comprises 106 known species in nine genera including 30 *Eresus* species. *Eresus* spiders have been reported in 52 locations in South Korea, excluding island regions, since they were first observed on Mt. Apsan in Daegu-si (Paik 1937) and they are generally known to be distributed throughout the Korean Peninsula(Namkung 1964, Paik 1978, Paik 1979, Namkung 1980, Kim 1985, Namkung 1986, Kim and Yoo 1996, Kim et al. 2015, Kim and Kim 2017, Kim 2021). Until now, due to their external morphological similarity, all *Eresus* spiders found in South Korea have been taxonomically identified as *E. kollari* (Paik 1978, Namkung 2001, Namkung 2003, Kim 2021). *Eresus kollari* is categorised as Endangered (EN) in the 2024 Red Data Book of the Republic of Korea because its distribution and population have continuously decreased due to the loss of grasslands and sand dunes, human activities, such as tourism and grave visiting and collection by the general public for hobby purposes (National Institute of Biological Resources (NIBR) 2024).

**Figure 1.**
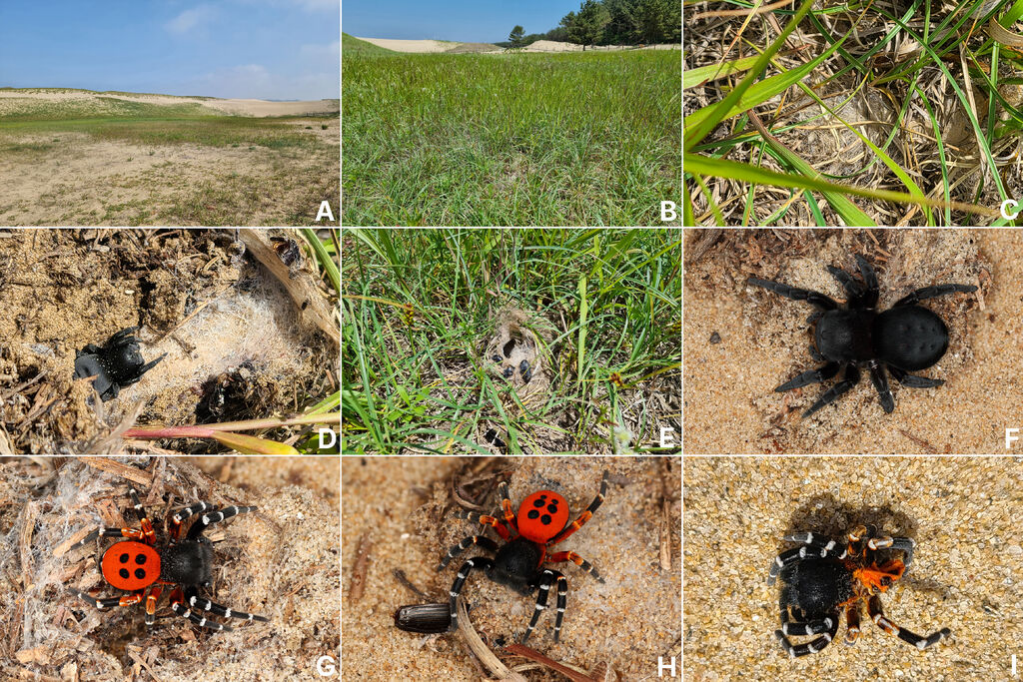
*Eresus granosus* Simon, 1895. **A** Habitat (Taean-gun, Chungcheongnam-do); **B** Microhabitat; **C** Exposed upper net on the ground; **D** Female with nest; **E** Beetle skeletons preyed upon at the entrance of the nest; **F** Female habitus; **G** Male habitus (four-spotted abdomen); **H** Ditto (six-spotted abdomen); **I** Dried-up carcass of a male on the hot sand.

**Figure 2.**
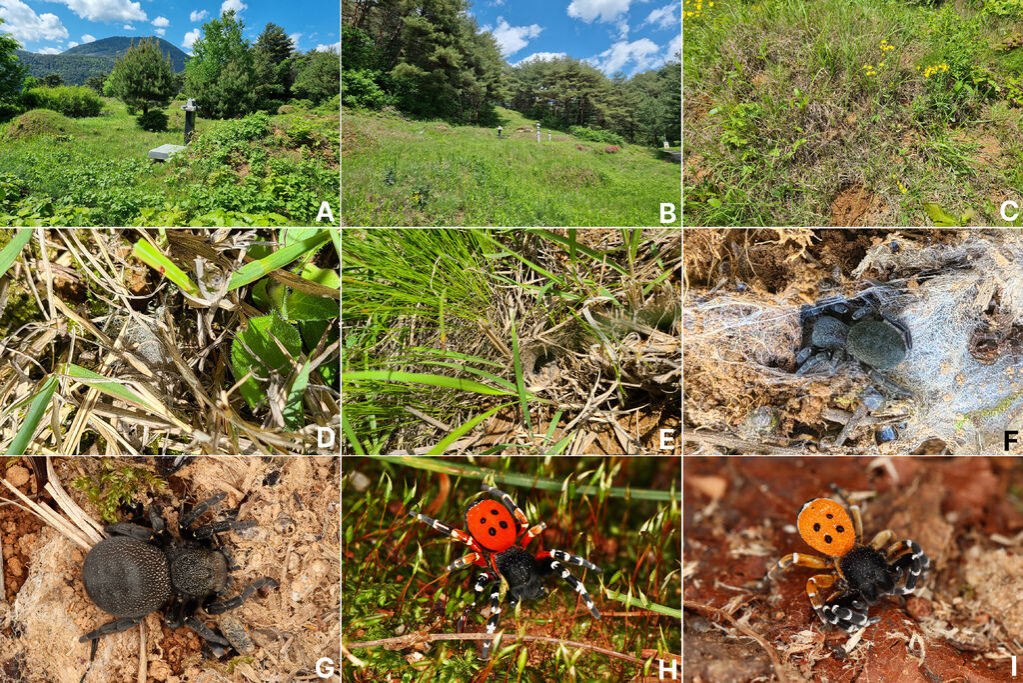
*Eresus kollari* Rossi, 1846. **A** Habitat (Jeongseon-gun, Gangwon-do); **B** Ditto (Pyeongchang-gun, Gangwon-do); **C** Microhabitat; **D** Exposed upper net on the ground; **E** Entrance of the nest; **F** Female with nest; **G** Female habitus; **H** Male habitus (red-coloured abdomen); **I** Ditto (orange-coloured abdomen).

The authors conducted an intensive survey of 46 out of the 52 locations where the *Eresus* spiders were previously known to distribute and were able to collect *Eresus* species from three regions: Taean-gun in Chungcheongnam-do and Jeongseon-gun and Pyeongchang-gun in Gangwon-do (Figs 1, 2). Based on a morphological taxonomic review, they confirmed that the *Eresus* spiders distributed in South Korea belong to two species: *E. kollari* and *E. granosus* Simon, 1895. Although no reliable and detailed taxonomic description of *E. granosus* has been available, a brief description, along with information on its habitat, distribution in China and photographs of the male and female genitalia, was recently provided (Zhang and Wang 2017). In the current paper, we describe two *Eresus* species, *E. kollari* and *E. granosus*, with a detailed description, morphological illustrations and ecological photographs from South Korea.

## Materials and methods

All specimens were collected by hands and preserved in 98% ethyl alcohol and external morphology was examined under a Leica S8APO stereomicroscope (Singapore). Images were captured with a Dhyana 400DC zoom digital camera (China), mounted on a Leica S8APO and assembled using Helicon Focus 8.2.0 image stacking software (Khmelik et al. 2006). Measurements of body parts were made with an ocular micrometer and are recorded in millimetres. Internal genitalia of females were removed and treated in 10% potassium hydroxide (KOH) for two hours before illustration. Legs and palp measurements are shown as: Total length (femur, patella, tibia, metatarsus, tarsus). Morphological terminology follows established usage in previous studies (Řezáč et al. 2008, Krejčí et al. 2015). The specimens examined are deposited in the National Institute of Biological Resources (NIBR), Incheon and Konkuk University (KKU), Seoul, Korea. The following abbreviations are used in the descriptions: **AER** = anterior eye row, **B** = bar, **C** = conductor, **CD** = copulatory duct, **E** = embolus, **F** = fissure, **FD** = fertilisation duct, **G** = groove, **L** = lamella, **PER** = posterior eye row, **S** = spermatheca, **SD** = sperm duct, **Sh** = shoulder, **T** = tegulum, **TT** = terminal tooth.

Total genomic DNA was extracted from spider legs or whole bodies in the case of small individuals, using a modified CTAB protocol (Zeh et al. 1992). Tissue samples were homogenised in CTAB buffer using a disposable pestle and 1.5 ml microcentrifuge tube. DNA was purified with two washes of chloroform: isoamyl alcohol (24:1) and the resulting pellet was air-dried at room temperature before being re-suspended in 50 µl of 0.1× TE buffer. DNA quality was assessed by electrophoresis on a 1% agarose gel and concentrations were measured using a Qubit IV Fluorometer (Invitrogen, Waltham, MA, USA). The mitochondrial cytochrome c oxidase subunit I (COI) region was amplified using primers LCO1490 and HC02198 (Folmer et al. 1994). PCR was carried out in a 20 µl reaction volume using HS Prime Taq Premix (GeNet Bio), containing 2 µl of genomic DNA and 0.5 pmol of each primer. The thermal cycling profile included an initial denaturation at 94°C for 5 minutes; 35 cycles of denaturation at 94°C for 30 seconds, annealing at 55°C for 30 seconds and extension at 72°C for 30 seconds; followed by a final extension at 72°C for 2 minutes. PCR products were confirmed by gel electrophoresis and sequenced by Macrogen. Raw sequences were manually edited using Geneious Prime (v.2025.0.3; Biomatters Ltd.). For comparative analysis, 13 *Eresus* and two *Loureedia* accessions were downloaded from GenBank. Accession numbers for each are listed in Figs. 5 and 6. All sequences were aligned using the Geneious Alignment algorithm in Geneious Prime and alignments were manually curated. A Neighbour-Joining (NJ) and Maximum Likelihood trees were constructed using Geneious Prime (v.2025.0.3). The population acronyms are used in the DNA Barcodes and Figures: **JS** = Jeongseon-gun, **PC** = Pyeongchang-gun, **TA** = Taean-gun.

## Taxon treatments

### Eresus granosus

Simon, 1895

0DF3D7A1-997F-5D2C-85FB-55BA024DEBF5

#### Materials

- **Type status:** Other material. **Occurrence:** catalogNumber: TTQXIV0000000229; recordedBy: Sue Yeon Lee, Chang Moon Jang & Seung Tae Kim; individualCount: 1; sex: male; lifeStage: adult; occurrenceID: 4886E523-BF7B-5D46-A662-2807D51EFD0C; **Taxon:** scientificName: *Eresus granosus*; kingdom: Animalia; phylum: Arthropoda; class: Arachnida; order: Araneae; family: Eresidae; **Location:** country: South Korea; stateProvince: Chungcheongbuk-do; municipality: Taean-gun; locality: Sindu-ri, Wonbuk-myeon; verbatimElevation: 37 m; verbatimCoordinates: 36°50'36.7"N 126°11'51.6"E; **Identification:** identifiedBy: Seung Tae Kim; **Event:** samplingProtocol: hand collecting; eventDate: 23-05-2024; habitat: coastal sand dune; **Record Level:** institutionCode: National Institute of Biological resources (NIBR)
- **Type status:** Other material. **Occurrence:** catalogNumber: KKU-Ere-20240623-01; recordedBy: Sue Yeon Lee, Chang Moon Jang & Seung Tae Kim; individualCount: 2; sex: male; lifeStage: adult; occurrenceID: 170EF2F0-268E-5B63-832A-F6956C2097F2; **Taxon:** scientificName: *Eresus granosus*; kingdom: Animalia; phylum: Arthropoda; class: Arachnida; order: Araneae; family: Eresidae; **Location:** country: South Korea; stateProvince: Chungcheongbuk-do; municipality: Taean-gun; locality: Sindu-ri, Wonbuk-myeon; verbatimElevation: 37 m; verbatimCoordinates: 36°50'36.7"N 126°11'51.6"E; **Identification:** identifiedBy: Seung Tae Kim; **Event:** samplingProtocol: hand collecting; eventDate: 23-05-2024; habitat: coastal sand dune; **Record Level:** institutionCode: Konkuk University (KKU)
- **Type status:** Other material. **Occurrence:** catalogNumber: TTQXIV0000000230; recordedBy: Sue Yeon Lee, Chang Moon Jang & Seung Tae Kim; individualCount: 1; sex: female; lifeStage: adult; occurrenceID: 4822F3C9-334E-5029-AD0F-0AEDF0014170; **Taxon:** scientificName: *Eresus granosus*; kingdom: Animalia; phylum: Arthropoda; class: Arachnida; order: Araneae; family: Eresidae; **Location:** country: South Korea; stateProvince: Chungcheongbuk-do; municipality: Taean-gun; locality: Sindu-ri, Wonbuk-myeon; verbatimElevation: 37 m; verbatimCoordinates: 36°50'36.7"N 126°11'51.6"E; **Identification:** identifiedBy: Seung Tae Kim; **Event:** samplingProtocol: hand collecting; eventDate: 23-05-2024; habitat: coastal sand dune; **Record Level:** institutionCode: National Institute of Biological resources (NIBR)
- **Type status:** Other material. **Occurrence:** catalogNumber: KKU-Ere-20240623-02; recordedBy: Sue Yeon Lee, Chang Moon Jang & Seung Tae Kim; individualCount: 2; sex: female; lifeStage: adult; occurrenceID: 4297BD0A-582A-54EF-9127-2B6A2978AC8C; **Taxon:** scientificName: *Eresus granosus*; kingdom: Animalia; phylum: Arthropoda; class: Arachnida; order: Araneae; family: Eresidae; **Location:** country: South Korea; stateProvince: Chungcheongbuk-do; municipality: Taean-gun; locality: Sindu-ri, Wonbuk-myeon; verbatimElevation: 37 m; verbatimCoordinates: 36°50'36.7"N 126°11'51.6"E; **Identification:** identifiedBy: Seung Tae Kim; **Event:** samplingProtocol: hand collecting; eventDate: 23-05-2024; habitat: coastal sand dune; **Record Level:** institutionCode: Konkuk University (KKU)

#### Description

**Figure 3.**
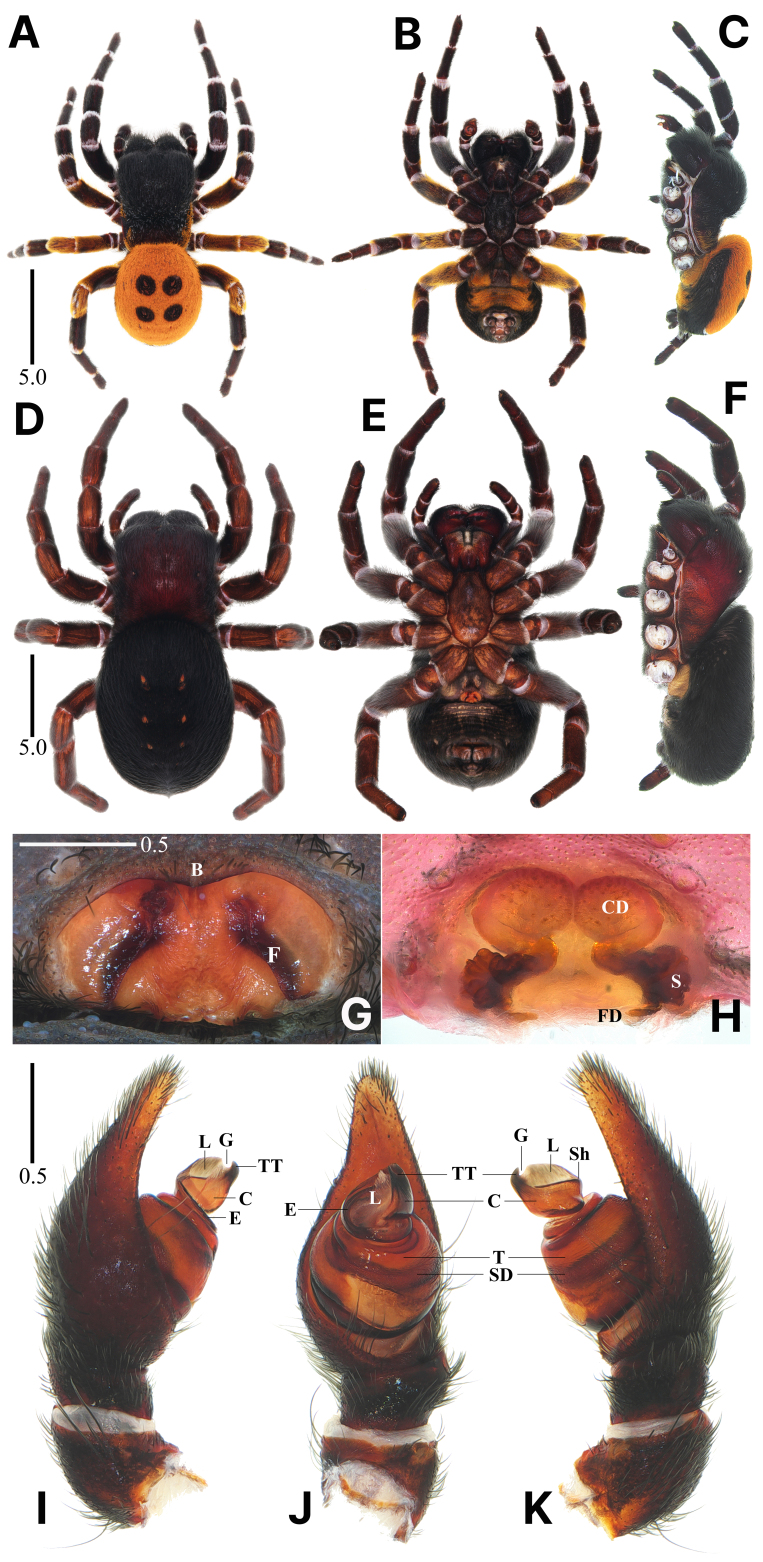
*Eresus granosus* Simon, 1895. **A**–**C** Male habitus, dorsal, ventral and lateral views; **D**–**F** Female habitus, dorsal, ventral and lateral views; **G** Epigyne, ventral view; **H** Internal genitalia, dorsal view; **I**–**K** Palpus, prolateral, ventral and retrolateral views (B = bar, C = conductor, CD = copulatory duct, E = embolus, F = fissure, FD = fertilisation duct, G = groove, L = lamella, S = spermatheca, SD = sperm duct, Sh = shoulder, T = tegulum, TT = terminal tooth). Scale bars in mm.

**Male.** Habitus as in Fig. 3A–C. Total length 9.91. Carapace: 5.46 long/3.62 wide/2.94 high. Eyes: AER 0.84, PER 2.98. Endite: 1.36 long/0.81 wide. Labium: 1.04 long/0.73 wide. Sternum: 2.74 long/1.71 wide. Legs: I 11.24 (3.62, 1.65, 1.97, 2.11, 1.89), II 9.78 (3.06, 1.56, 1.62, 1.88, 1.66), III 8.72 (2.95, 1.57, 1.27, 1.64, 1.29), IV 11.36 (3.63, 1.90, 2.08, 2.17, 1.59). Palp: 3.42 (1.39, 0.60, 0.28, - , 1.15). Abdomen: 5.37 long/4.58 wide.

Carapace black, rectangular, longer than wide; head region elevated, covered densely with black setae; thoracic region covered with black and white setae, orange setae lined along the margin; AER < PER (Fig. 3A and C). Chelicerae stout, black, covered with black setae (Fig. 3A–C). Endite black, slightly curved, anterior edge blunt (Fig. 3B). Sternum nearly black, narrow, much longer than wide, covered densely with black and white setae, anterior edge truncated (Fig. 3B). Legs thick and strongly developed, covered densely with black, white and orange setae; mostly black, with femur II and femur and patella III, IV orange; joints with white annuli; leg formula IV-I-II-III (Fig. 3A–C). Abdomen orange, with four or six black, round spots, anterior part protruding above the thoracic region (Fig. 3A and C). Palpus (Fig. 3I–K): tegulum round; embolus rotating clockwise along the top of the tegulum; conductor sclerotised, wider than long, with a prominent shoulder; terminal tooth sclerotised, slightly incurvated towards groove; groove U-shaped; lamella translucent with a feather-like edge, subequal with a terminal tooth in length.

**Female.** Habitus as in Fig. 3D–F. Total length 16.43. Carapace: 9.14 long/6.25 wide/ 3.83. Eyes: AER 1.07, PER 4.05. Endite: 2.12 long/1.31 wide. Labium: 1.54 long/1.10 wide. Sternum: 4.49 long/2.23 wide. Legs: I 15.07 (4.91, 2.59, 2.55, 2.68, 2.34), II 13.39 (4.32, 2.53, 2.07, 2.48, 1.99), III 11.71 (4.19, 2.36, 1.82, 1.92, 1.42), IV 16.10 (5.63, 2.86, 3.05, 2.72, 1.84). Palp: 6.23 (2.25, 1.27, 0.88, - , 1.83). Abdomen: 10.40 long/8.40 wide. Epigynum: 1.24 wide.

Carapace dark reddish-black, rectangular, longer than wide, covered densely with black setae; head region elevated; AER < PER (Fig. 3D and F). Chelicerae stout, reddish-black, covered with black setae (Fig. 3D–F). Endite reddish-black, straight, anterior edge angular, truncated (Fig. 3E). Sternum reddish-brown mottled with black, narrow, much longer than wide, anterior edge truncated, covered densely with black setae (Fig. 3E). Legs thick and strongly developed, covered densely with black setae; blackish-brown, dorsum with one or two reddish-brown streaks; leg formula IV-I-II-III (Fig. 3D–F). Abdomen uniformly black, with three pairs of muscle impressions, dorsum flat, anterior part protruding above the thoracic region (Fig. 3D and F). Epigyne (Fig. 3G): elliptical with a sclerotised margin, anterior bar wide, slightly depressed, wider than long, fissure anteriorly incurvated, slightly towards the centre. Internal genitalia (Fig. 3H): copulatory ducts large, translucent, contiguous, anterior section circular; spermathecae very distinctly lobated, reaching further laterally than copulatory ducts.

#### Habitat

This species is found to inhabit the western coastal sand dunes in South Korea (Fig. 1A). The spider builds a silken tunnel, about 15–20 cm in length, underground in the sandy soil between coastal plants, such as strand sedges (*Carex pumila* Thunb.) (Cyperaceae) (Fig. 1B). A sheet web is attached above the entrance of the silken tube, which is connected to the ground (Fig. 1C). Sometimes, the exoskeletons of beetles, such as Carabidae and Tenebrionidae spp. (Insecta, Coleoptera), which have been preyed upon by the spider, can be found attached to the entrance (Fig. 1E).

#### Distribution

Russia (West Siberia), China, South Korea (new record) (World Spider Catalog 2025).

#### DNA Barcodes

***TA1***

AAAATCAAAATAAATGCTGAAATAAAATAGGATCTCCTCCCCCAGCAGGATCAAAAAACGATGTATTAAAATTTCGATCAGTTAATAATATTGTAATAGCACCCGCTAAAACAGGTAAAGATAATAATAATAATACCGCAGTAATTAAAACAGATCAAACAAATAATGGTACCTTCTCTATAGTTATTCCATATGAACGTATATTAAGTACAGTAGTAATAAAATTAATAGCCCCCATAATAGAAGAAGCCCCAGCTAAATGTAATGAAAAAATGGCAAAATCTACTGATCTCCCCGCATGACCCATTAATGAGGCTAAAGGAGGGTAAACAGTTCACCCTGTCCCTACACCCATTTCTACTATAGAAGATATAAATAATATAAACAATGAAGGAGGCAATAACCAAAAACTCAAATTATTTATTCGAGGAAAAGCTATATCAGGTGCTCCTAATATTAAAGGAACCAATCAATTCCCAAACCCACCAATTATAATTGGTATAACTATAAAAAAAATTATCACAAAAGCATGAGCAGTAACAACAACATTATACAAATGATCATCTCCTAATAATCTCCCAGATTGTCCTAATTCTGTTCGAATAATTATTCTTATTGAAGTTCCAACTATAGCTGATCAAGCTCCAAAAATTAAATACAATGTTCCAA (GenBank accession number PV785319)

***TA2***

AAAATCAAAATAAATGCTGAAATAAAATAGGATCTCCTCCCCCAGCAGGATCAAAAAACGATGTATTAAAATTTCGATCAGTTAATAATATTGTAATAGCACCCGCTAAAACAGGTAAAGATAATAATAATAATACCGCAGTAATTAAAATAGATCAAACAAATAATGGTACCTTCTCTATAGTTATTCCATATGAACGTATATTAAGTACAGTAGTAATAAAATTAATAGCCCCCATAACAGAAGAAGCCCCAGCTAAATGTAATGAAAAAATGGCAAAATCTACTGATCTCCCCGCATGACCTATTAATGAGGCTAAAGGAGGGTAAACAGTTCACCCTGTCCCTACACCCATTTCTACTATAGAAGATATAAATAATATAAACAATGAAGGAGGCAATAACCAAAAACTCAAATTATTTATTCGAGGAAAAGCTATATCAGGTGCTCCTAATATTAAAGGAACCAATCAATTCCCAAACCCACCAATTATAATTGGTATAACTATAAAAAAAATTATCACAAAAGCATGAGCAGTAACAACAACATTATACAAATGATCATCTCCTAATAATCTCCCAGATTGTCCTAATTCTGTTCGAATAATTATTCTTATTGAAGTCCCAACTATAGCTGATCAAGCTCCAAAAATTAAATACAATGTTCCAA (GenBank accession number PV785320)

***TA4***

AAAATCAAAATAAATGCTGAAATAAAATAGGATCTCCTCCCCCAGCAGGATCAAAAAACGATGTATTAAAATTTCGATCAGTTAATAATATTGTAATAGCACCCGCTAAAACAGGTAAAGATAATAATAATAATACCGCAGTAATTAAAATAGATCAAACAAATAATGGTACCTTCTCTATAGTTATTCCATATGAACGTATATTAAGTACAGTAGTAATAAAATTAATAGCCCCCATAACAGAAGAAGCCCCAGCTAAATGTAATGAAAAAATGGCAAAATCTACTGATCTCCCCGCATGACCTATTAATGAGGCTAAAGGAGGGTAAACAGTTCACCCTGTCCCTACACCCATTTCTACTATAGAAGATATAAATAATATAAACAATGAAGGAGGCAATAACCAAAAACTCAAATTATTTATTCGAGGAAAAGCTATATCAGGTGCTCCTAATATTAAAGGAACCAATCAATTCCCAAACCCACCAATTATAATTGGTATAACTATAAAAAAAATTATCACAAAAGCATGAGCAGTAACAACAACATTATACAAATGATCATCTCCTAATAATCTCCCAGATTGTCCTAATTCTGTTCGAATAATTATTCTTATTGAAGTCCCAACTATAGCTGATCAAGCTCCAAAAATTAAATACAATGTTCCAA (GenBank accession number PV785321)

***TA5***

AAAATCAAAATAAATGCTGAAATAAAATAGGATCTCCTCCCCCAGCAGGATCAAAAAACGATGTATTAAAATTTCGATCAGTTAATAATATTGTAATAGCACCCGCTAAAACAGGTAAAGATAATAATAATAATACCGCAGTAATTAAAACAGATCAAACAAATAATGGTACCTTCTCTATAGTTATTCCATATGAACGTATATTAAGTACAGTAGTAATAAAATTAATAGCCCCCATAATAGAAGAAGCCCCAGCTAAATGTAATGAAAAAATGGCAAAATCTACTGATCTCCCCGCATGACCCATTAATGAGGCTAAAGGAGGGTAAACAGTTCACCCTGTCCCTACACCCATTTCTACTATAGAAGATATAAATAATATAAACAATGAAGGAGGCAATAACCAAAAACTCAAATTATTTATTCGAGGAAAAGCTATATCAGGTGCTCCTAATATTAAAGGAACCAATCAATTCCCAAACCCACCAATTATAATTGGTATAACTATAAAAAAAATTATCACAAAAGCATGAGCAGTAACAACAACATTATACAAATGATCATCTCCTAATAATCTCCCAGATTGTCCTAATTCTGTTCGAATAATTATTCTTATTGAAGTTCCAACTATAGCTGATCAAGCTCCAAAAATTAAATACAATGTTCCAA (GenBank accession number PV785322)

***TA6***

AAAATCAAAATAAATGCTGAAATAAAATAGGATCTCCTCCCCCAGCAGGATCAAAAAACGATGTATTAAAATTTCGATCAGTTAATAATATTGTAATAGCACCCGCTAAAACAGGTAAAGATAATAATAATAATACCGCAGTAATTAAAATAGATCAAACAAATAATGGTACCTTCTCTATAGTTATTCCATATGAACGTATATTAAGTACAGTAGTAATAAAATTAATAGCCCCCATAACAGAAGAAGCCCCAGCTAAATGTAATGAAAAAATGGCAAAATCTACTGATCTCCCCGCATGACCTATTAATGAGGCTAAAGGAGGGTAAACAGTTCACCCTGTCCCTACACCCATTTCTACTATAGAAGATATAAATAATATAAACAATGAAGGAGGCAATAACCAAAAACTCAAATTATTTATTCGAGGAAAAGCTATATCAGGTGCTCCTAATATTAAAGGAACCAATCAATTCCCAAACCCACCAATTATAATTGGTATAACTATAAAAAAAATTATCACAAAAGCATGAGCAGTAACAACAACATTATACAAATGATCATCTCCTAATAATCTCCCAGATTGTCCTAATTCTGTTCGAATAATTATTCTTATTGAAGTCCCAACTATAGCTGATCAAGCTCCAAAAATTAAATACAATGTTCCAA (GenBank accession number PV785323)

### Eresus kollari

Rossi, 1846

6DD91DF8-713C-5FE9-9497-E0DA7FF5E674

#### Materials

- **Type status:** Other material. **Occurrence:** catalogNumber: TTQXIV0000000231; recordedBy: Sue Yeon Lee, Chang Moon Jang & Seung Tae Kim; individualCount: 1; sex: male; lifeStage: adult; occurrenceID: C264EF20-34D2-53D6-8DF4-10166AE878FE; **Taxon:** scientificName: *Eresus kollari*; kingdom: Animalia; phylum: Arthropoda; class: Arachnida; order: Araneae; family: Eresidae; **Location:** country: South Korea; stateProvince: Gangwon State; municipality: Jeongseon-gun; locality: Nampyeong-ri, Bukpyeong-myeon; verbatimElevation: 366 m; verbatimCoordinates: 37°26'21.8"N 128°39'51.0"E; **Identification:** identifiedBy: Seung Tae Kim; **Event:** samplingProtocol: hand collecting; eventDate: 03-10-2023; habitat: grassy grave; **Record Level:** institutionCode: National Institute of Biological resources (NIBR)
- **Type status:** Other material. **Occurrence:** catalogNumber: KKU-Ere-20231003-01; recordedBy: Sue Yeon Lee, Chang Moon Jang & Seung Tae Kim; individualCount: 1; sex: male; lifeStage: adult; occurrenceID: 095BD786-A8E0-5F50-BA85-9B2C99801B92; **Taxon:** scientificName: *Eresus kollari*; kingdom: Animalia; phylum: Arthropoda; class: Arachnida; order: Araneae; family: Eresidae; **Location:** country: South Korea; stateProvince: Gangwon State; municipality: Jeongseon-gun; locality: Nampyeong-ri, Bukpyeong-myeon; verbatimElevation: 366 m; verbatimCoordinates: 37°26'21.8"N 128°39'51.0"E; **Identification:** identifiedBy: Seung Tae Kim; **Event:** samplingProtocol: hand collecting; eventDate: 03-10-2023; habitat: grassy grave; **Record Level:** institutionCode: Konkuk University (KKU)
- **Type status:** Other material. **Occurrence:** catalogNumber: TTQXIV0000000232; recordedBy: Sue Yeon Lee, Chang Moon Jang & Seung Tae Kim; individualCount: 1; sex: female; lifeStage: adult; occurrenceID: 4F7E3E76-C42A-5070-B15B-DE6E5F28D561; **Taxon:** scientificName: *Eresus kollari*; kingdom: Animalia; phylum: Arthropoda; class: Arachnida; order: Araneae; family: Eresidae; **Location:** country: South Korea; stateProvince: Gangwon State; municipality: Jeongseon-gun; locality: Nampyeong-ri, Bukpyeong-myeon; verbatimElevation: 366 m; verbatimCoordinates: 37°26'21.8"N 128°39'51.0"E; **Identification:** identifiedBy: Seung Tae Kim; **Event:** samplingProtocol: hand collecting; eventDate: 08-05-2024; habitat: grassy grave; **Record Level:** institutionCode: National Institute of Biological resources (NIBR)
- **Type status:** Other material. **Occurrence:** catalogNumber: KKU-Ere-20230508-01; recordedBy: Sue Yeon Lee, Chang Moon Jang & Seung Tae Kim; individualCount: 2; sex: female; lifeStage: adult; occurrenceID: F333E982-0AEC-52E3-8A4C-EF4D596C4409; **Taxon:** scientificName: *Eresus kollari*; kingdom: Animalia; phylum: Arthropoda; class: Arachnida; order: Araneae; family: Eresidae; **Location:** country: South Korea; stateProvince: Gangwon State; municipality: Jeongseon-gun; locality: Nampyeong-ri, Bukpyeong-myeon; verbatimElevation: 366 m; verbatimCoordinates: 37°26'21.8"N 128°39'51.0"E; **Identification:** identifiedBy: Seung Tae Kim; **Event:** samplingProtocol: hand collecting; eventDate: 08-05-2024; habitat: grassy grave; **Record Level:** institutionCode: Konkuk University (KKU)
- **Type status:** Other material. **Occurrence:** catalogNumber: KKU-Ere-20231003-02; recordedBy: Sue Yeon Lee, Chang Moon Jang & Seung Tae Kim; individualCount: 2; sex: female; lifeStage: adult; occurrenceID: 2A109105-44ED-5CC2-9EDC-A273EED14A09; **Taxon:** scientificName: *Eresus kollari*; kingdom: Animalia; phylum: Arthropoda; class: Arachnida; order: Araneae; family: Eresidae; **Location:** country: South Korea; stateProvince: Gangwon State; municipality: Pyeongchang-gun; locality: Jongbu-ri, Pyeongchang-eup; verbatimElevation: 391 m; verbatimCoordinates: 37°21'42.8"N 128°23'44.8"E; **Identification:** identifiedBy: Seung Tae Kim; **Event:** samplingProtocol: hand collecting; eventDate: 03-10-2023; habitat: grassy grave; **Record Level:** institutionCode: Konkuk University (KKU)

#### Description

**Figure 4.**
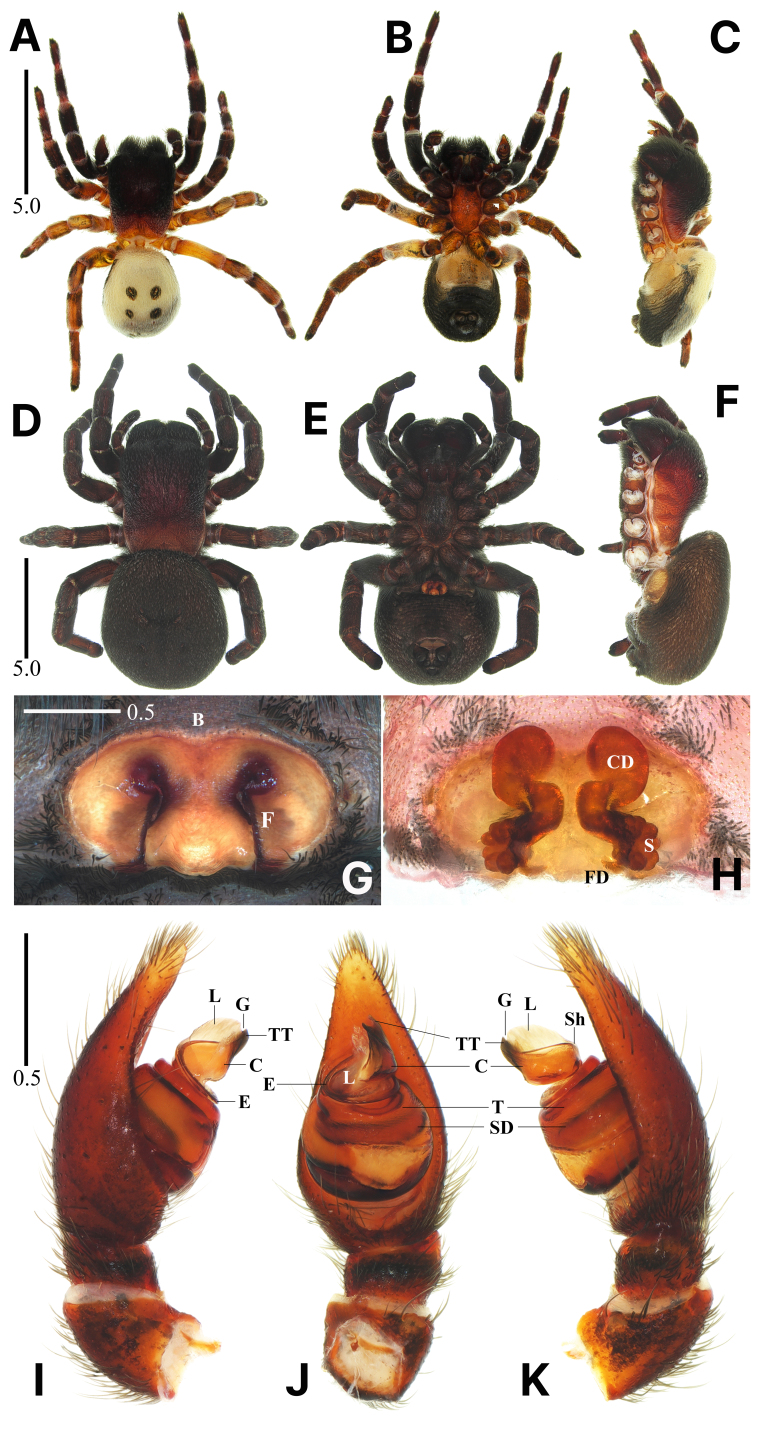
*Eresus kollari* Rossi, 1846. **A**–**C** Male habitus, dorsal, ventral and lateral views; **D**–**F** Female habitus, dorsal, ventral and lateral views; **G** Epigyne, ventral view; **H** Internal genitalia, dorsal view; **I**–**K** Palpus, prolateral, ventral and retrolateral views (B = bar, C = conductor, CD = copulatory duct, E = embolus, F = fissure, FD = fertilisation duct, G = groove, L = lamella, S = spermatheca, SD = sperm duct, Sh = shoulder, T = tegulum, TT = terminal tooth). Scale bars in mm.

**Male.** Habitus as in Fig. 4A–C. Total length 8.23. Carapace: 3.78 long/2.72 wide/2.00 high. Eyes: AER 0.68, PER 2.16. Endite: 1.10 long/0.60 wide. Labium: 0.85 long/0.60 wide. Sternum: 2.13 long/1.39 wide. Legs: I 7.95 (2.34, 1.29, 1.38, 1.51, 1.43), II 6.63 (1.95, 1.10, 1.22, 1.18, 1.18), III 5.68 (1.73, 1.10, 0.97, 1.01, 0.90), IV 8.19 (2.65, 1.36, 1.60, 1.50, 1.08). Palp: 3.06 (1.23, 0.55, 0.22, - , 1.06). Abdomen: 4.20 long/3.38 wide.

Carapace black, rectangular, longer than wide; head region elevated, covered densely with black and white setae; thoracic region covered with black and white setae, posterior part dark orange; AER < PER (Fig. 4A and C). Chelicerae stout, black, covered with black setae (Fig. 4A–C). Endite black, slightly curved, anterior edge blunt (Fig. 4B). Sternum dark orange mottled with black, narrow, much longer than wide, covered densely with black and white setae, anterior edge truncated (Fig. 4B). Legs thick and strongly developed, covered densely with black, white and orange setae; Legs I and II mostly black, femur II dark orange, joints orange with white annuli; legs III and IV turbid orange mottled with black, joints with white annuli; leg formula IV-I-II-III (Fig. 4A–C). Abdomen ivory, with four black, round spots, anterior part protruding above the thoracic region (Fig. 4A and C). Palpus (Fig. 4I–K): tegulum round; embolus rotating clockwise along the top of the tegulum; conductor sclerotised, wider than long, with a prominent shoulder; terminal tooth sclerotised, straight; groove small, V-shaped; lamella translucent with a feather-like edge, slightly longer than terminal tooth.

**Female.** Habitus as in Fig. 4D–F. Total length 12.89. Carapace: 6.11 long/4.24 wide/3.07 high. Eyes: AER 0.87, PER 3.09. Endite: 1.71 long/0.97 wide. Labium: 1.29 long/0.96 wide. Sternum: 3.28 long/2.01 wide. Legs: I 10.15 (3.04, 1.74, 1.74, 1.99, 1.64), II 9.08 (2.81, 1.65, 1.53, 1.51, 1.58), III 8.17 (2.79, 1.70, 1.32, 1.21, 1.15), IV 10.83 (3.69, 1.99, 2.01, 1.91, 1.23). Palp: 4.45 (1.54, 0.94, 0.62, - , 1.35). Abdomen: 6.52 long/6.20 wide. Epigynum: 1.35 wide.

Carapace blackish-brown, rectangular, longer than wide, covered densely with black and white setae; head region elevated; AER < PER (Fig. 4D and F). Chelicerae stout, blackish-brown, covered with black and white setae (Fig. 4D–F). Endite blackish-brown, straight, anterior edge angular, truncated (Fig. 4E). Sternum blackish-brown, medially black, narrow, much longer than wide, anterior edge truncated, covered densely with black setae (Fig. 4E). Legs thick and strongly developed, covered densely with black setae; blackish-brown, joints with white annuli; leg formula IV-I-II-III (Fig. 4D–F). Abdomen blackish-brown, with four pairs of muscle impressions, dorsum gently sloped, anterior part protruding above the thoracic region (Fig. 4D and F). Epigyne (Fig. 4G): elliptical with a sclerotised margin, anterior bar wide, slightly depressed, wider than long, fissure anteriorly incurvated, almost longitudinal. Internal genitalia (Fig. 3H): copulatory ducts large, translucent, anterior section elliptical, far from each other; spermathecae distinctly lobated, extended laterally to the copulatory ducts.

#### Habitat

This species is frequently found inhabiting tombs at hilly gravesites in South Korea (Fig. 2A and B). The spider builds a silken tunnel, about 15–20 cm in length, underground in the yellow soil between grasses, such as fine-leaf shae sedges (Carex humilis var. nana Ohwi) (Cyperaceae) and zoysia grass (*Zoysia japonica* Steud) (Poaceae) (Fig. 2C). A sheet web is attached above the entrance of the silken tube, which is connected to the ground (Fig. 2D and E).

#### Distribution

Europe, Turkey, Caucasus, Iran, China, South Korea, Russia (to Far East), Central Asia (World Spider Catalog 2025).

#### DNA Barcodes

***JS1***

AAAATCAAAATAAATGTTGAAATAAAATAGGATCTCCTCCCCCAGCAGGATCAAAAAACGATGTATTAAAATTTCGATCAGTTAATAATATTGTAATAGCACCCGCTAAAACAGGTAAAGATAACAACAATAATACCGCAGTAATTAAAACAGATCAAACAAATAATGATACCTTCTCCATTGTTATTCCATATGAACGTATATTAATTACAGTTGTAATAAAATTAATAGCCCCCATAATAGAAGAAGCCCCAGCTAAATGTAATGAAAAAATAGCAAAATCTACTGATCTCCCCGCATGACCTATTAATGACGTTAAAGGAGGATAAACAGTTCACCCTGTCCCTACACCCATTTCTACTATAGAAGACATAAATAATATAAACAATGAAGGAGGTAATAACCAAAAACTTAAATTATTTATTCGAGGAAAAGCTATATCAGGTGCCCCTAATATTAAAGGAACCAATCAATTCCCAAACCCTCCAATTATAATTGGTATAACTATAAAAAAAATTATAACAAAAGCATGAGCAGTAACAATAACATTATATAAATGATCATCTCCTAATAATCTCCCAGATTGTCCTAATTCCGTTCGAATAATTATTCTTATTGAAGTTCCAACTATAGCTGATCAAGCTCCAAAAATTAAATACAACGTTCCAA (GenBank accession number PV785314)

***JS2***

AAAATCAAAATAAATGTTGAAATAAAATAGGATCTCCTCCCCCAGCAGGATCAAAAAACGATGTATTAAAATTTCGATCAGTTAATAATATTGTAATAGCACCCGCTAAAACAGGTAAAGATAACAACAATAATACCGCAGTAATTAAAACAGATCAAACAAATAATGATACCTTCTCCATTGTTATTCCATATGAACGTATATTAATTACAGTTGTAATAAAATTAATAGCCCCCATAATAGAAGAAGCCCCAGCTAAATGTAATGAAAAAATAGCAAAATCTACTGATCTCCCCGCATGACCTATTAATGACGCTAAAGGAGGATAAACAGTTCACCCTGTCCCTACACCCATTTCTACTATAGAAGACATAAATAATATAAACAATGAAGGAGGTAATAACCAAAAACTTAAATTATTTATTCGAGGAAAAGCTATATCAGGTGCCCCTAATATTAAAGGAACCAATCAATTCCCAAACCCTCCAATTATAATTGGTATAACTATAAAAAAAATTATAACAAAAGCATGAGCAGTAACAATAACATTATATAAATGATCATCTCCTAATAATCTCCCAGATTGTCCTAATTCCGTTCGAATAATTATTCTTATTGAAGTTCCAACTATAGCTGATCAAGCTCCAAAAATTAAATACAACGTTCCAA (GenBank accession number PV785315)

***JS3***

AAAATCAAAATAAATGTTGAAATAAAATAGGATCTCCTCCCCCAGCAGGATCAAAAAACGATGTATTAAAATTTCGATCAGTTAATAATATTGTAATAGCACCCGCTAAAACAGGTAAAGATAACAACAATAATACCGCAGTAATTAAAACAGATCAAACAAATAATGATACCTTCTCCATTGTTATTCCATATGAACGTATATTAATTACAGTTGTAATAAAATTAATAGCCCCCATAATAGAAGAAGCCCCAGCTAAATGTAATGAAAAAATAGCAAAATCTACTGATCTCCCCGCATGACCTATTAATGACGTTAAAGGAGGATAAACAGTTCACCCTGTCCCTACACCCATTTCTACTATAGAAGACATAAATAATATAAACAATGAAGGAGGTAATAACCAAAAACTTAAATTATTTATTCGAGGAAAAGCTATATCAGGTGCCCCTAATATTAAAGGAACCAATCAATTCCCAAACCCTCCAATTATAATTGGTATAACTATAAAAAAAATTATAACAAAAGCATGAGCAGTAACAATAACATTATATAAATGATCATCTCCTAATAATCTCCCAGATTGTCCTAATTCCGTTCGAATAATTATTCTTATTGAAGTTCCAACTATAGCTGATCAAGCTCCAAAAATTAAATACAACGTTCCAA (GenBank accession number PV785316)

***PC1***

AAAATCAAAATAAATGTTGAAATAAAATAGGATCTCCTCCCCCAGCAGGATCAAAAAACGATGTATTAAAATTTCGATCAGTTAATAATATTGTAATAGCACCCGCTAAAACAGGTAAAGATAACAACAATAATACCGCAGTAATTAAAACAGATCAAACAAATAATGATACCTTCTCCATTGTTATTCCATATGAACGTATATTAATTACAGTTGTAATAAAATTAATAGCCCCCATAATAGAAGAAGCCCCAGCTAAATGTAATGAAAAAATAGCAAAATCTACTGATCTCCCCGCATGACCTATTAATGACGCTAAAGGAGGATAAACAGTTCACCCTGTCCCTACACCCATTTCTACTATAGAAGACATAAATAATATAAACAATGAAGGAGGTAATAACCAAAAACTTAAATTATTTATTCGAGGAAAAGCTATATCAGGTGCCCCTAATATTAAAGGAACCAATCAATTCCCAAACCCTCCAATTATAATTGGTATAACTATAAAAAAAATTATAACAAAAGCATGAGCAGTAACAATAACATTATATAAATGATCATCTCCTAATAATCTCCCAGATTGTCCTAATTCCGTTCGAATAATTATTCTTATTGAAGTTCCAACTATAGCTGATCAAGCTCCAAAAATTAAATACAACGTTCCAA (GenBank accession number PV785317)

***PC2***

AAATCAAAATAAATGTTGAAATAAAATAGGATCTCCTCCCCCAGCAGGATCAAAAAACGATGTATTAAAATTTCGATCAGTTAATAATATTGTAATAGCACCCGCTAAAACAGGTAAAGATAACAACAATAATACCGCAGTAATTAAAACAGATCAAACAAATAATGATACCTTCTCCATTGTTATTCCATATGAACGTATATTAATTACAGTTGTAATAAAATTAATAGCCCCCATAATAGAAGAAGCCCCAGCTAAATGTAATGAAAAAATAGCAAAATCTACTGATCTCCCCGCATGACCTATTAATGACGCTAAAGGAGGATAAACAGTTCACCCTGTCCCTACACCCATTTCTACTATAGAAGACATAAATAATATAAACAATGAAGGAGGTAATAACCAAAAACTTAAATTATTTATTCGAGGAAAAGCTATATCAGGTGCCCCTAATATTAAAGGAACCAATCAATTCCCAAACCCTCCAATTATAATTGGTATAACTATAAAAAAAATTATAACAAAAGCATGAGCAGTAACAATAACATTATATAAATGATCATCTCCTAATAATCTCCCAGATTGTCCTAATTCCGTTCGAATAATTATTCTTATTGAAGTTCCAACTATAGCTGATCAAGCTCCAAAAATTAAATACAACGTTCCAA (GenBank accession number PV785318)

## Discussion

**Figure 5.**
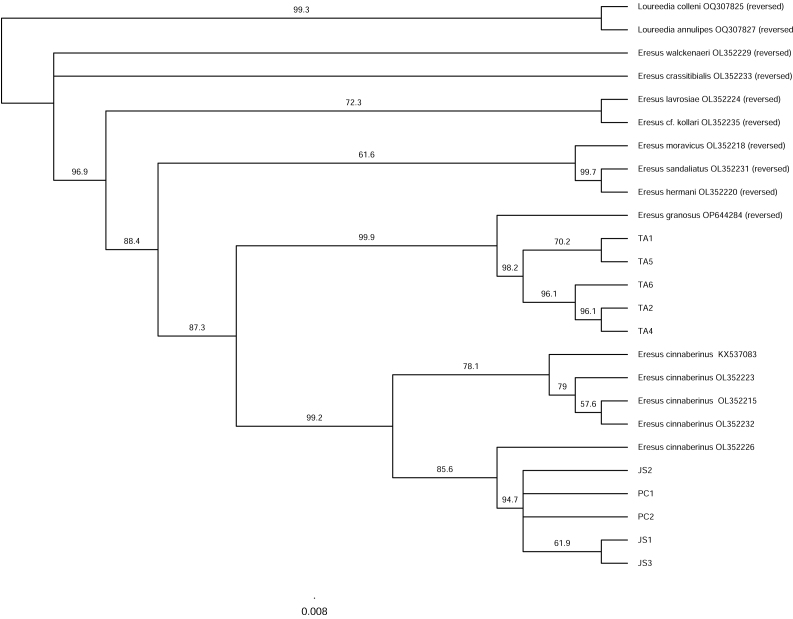
Neighbour-Joining tree inferred from mitochondrial CO1 gene. Node support values are based on 1,000 bootstrap replicates (JS = Jeongseon-gun, PC = Pyeongchang-gun, TA = Taean-gun. *Eresus cinnaberinus* is currently considered synonymous with *Eresus kollari*).

**Figure 6.**
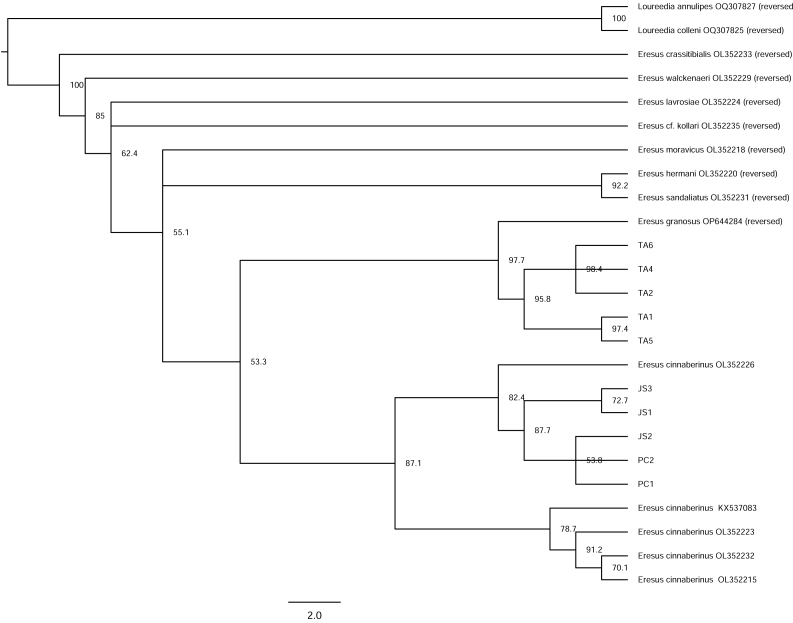
Maximum-Likelihood tree inferred from mitochondrial CO1 gene. Node support values are based on 1,000 bootstrap replicates (JS = Jeongseon-gun, PC = Pyeongchang-gun, TA = Taean-gun. *Eresus cinnaberinus* is currently considered synonymous with *Eresus kollari*).

Phylogenetic analyses, together with morphological distinctions, provide robust evidence that the single species of *Eresus* previously reported from South Korea comprises two distinct species. Both the Neighbour-Joining (NJ) and Maximum Likelihood (ML) trees, inferred from the mitochondrial CO1 region, support the separation of two *Eresus* species in South Korea (Figs 5, 6). The Taean (TA) population clustered with *E. granosus* from Xinjiang, China, whereas the Jeongseon (JS) and Pyeongchang (PC) populations clustered with *E. kollari* from Hungary.

## Supplementary Material

XML Treatment for Eresus granosus

XML Treatment for Eresus kollari
